# A comparative study on the monovalent and divalent cation separation of polymeric films and membranes from salt solutions under diffusion-dialysis

**DOI:** 10.3906/kim-2004-26

**Published:** 2020-08-18

**Authors:** Serkan ACAR, Hacer Yeşim CENGİZ, Ayça ERGÜN, Eymen KONYALI, Hüseyin DELİGÖZ

**Affiliations:** 1 Department of Chemical Engineering, Engineering Faculty, İstanbul University-Cerrahpaşa, İstanbul Turkey

**Keywords:** Ion separation, diffusion, polymeric films, nanofiltration membranes, separation factor

## Abstract

This study deals with selective separation of mono- and divalent cations from aqueous salt solutions using polymeric films based on polyethylene (PE) and polyamide6 (PA6), and two different commercial nanofiltration (NF) membranes. The diffusion rates (D) of ions (Na^+^ and Ca^2+^), separation factors (α) and ion rejections (R) of the films and NF membranes are examined comparatively as well as their surface morphology and hydrophilicity. It is observed that the diffusion rates of Na^+^ are in the range of 0.7–1.8 × 10^−8^cm^2^ .s^−1^ in the decreasing order of PE > NF90 > NF270 > PA6 while Ca^2+^ shows diffusion rates of 7.4–18.4 × 10^−8^ cm^2^ .s^−1^ in the increasing order of NF270 > NF90 ≈ PA6 > PE. Rejection values of the polymeric films and NF membranes against to Na^+^ and Ca^2+^ vary between 90% and 99.6%.The highest α(Ca^2+^/Na^+^) is found to be 20 for PA6 film. D, α, and R value of both polymeric films and NF membranes are strongly affected by the existence of osmosis during diffusion-dialysis and the sizes of hydrated sodiu and calcium ions. In conclusion, the film based on PA6 may be a good alternative for selective separation of mono- an divalent cations.

## 1. Introduction

Depending on the developments in material science and technology, various separation techniques have attracted great attention in the chemical industry. Some conventional separation techniques such as distillation, evaporatio and extraction are currently used in various industries including food [1, 2], beverage [3, 4], mining [5–7], etc. Recently, membrane technology offered a good alternative for the separation purposes in terms of good performance, economy, low energy consumption and easy adaptability to various applications. Based on the separation type, there are some different membrane processes such as microfiltration (MF), ultrafiltration (UF), nanofiltration (NF) and reverse osmosis (RO). For these processes, different polymer-based films were used as membranes because of the use of ceramic-based membranes were relatively limited. In the last a few decades different approaches were used for the development of appropriate separation materials. These are; insertion of metal oxides to polymeric membranes [8–10], designing nanocomposite structured polymeric membranes [11–13], thin film composite (TFC) membranes [14–16], liquid membranes [17], electromembranes [18,19]. In particular, the separation of some ions with low charge and small atomic diameter is really hard and hence it would be very useful to find good candidates for ion removal with high selectivity.

The use of polymeric films, its composites and polymer based membranes are more commonly applied in gas [20–25] or aqueous salt [26–33] separation. In gas separation, Kumar et al. investigated H_2_, N_2_ and CO_2_ separation capability of polystyrene (PS) based membrane including multiwalled carbon nanotube (MWCNT). It was observed that the permeability of H_2_ increased by the dispersion of MWCNT in PS compared to bare PS [25]. In another study, the membrane consisting of block copolymer of PS and poly (ethylene oxide) was employed for gas separation and high permeabilities for CO_2_, He, O_2_, CH_4_, N_2_ were described [22]. While Hartel et al. reported the use of PET membranes in separation of H_2_/CO_2_ gas mixtures [21], Kamakshi et al. studied the permeability of H_2_ through a porous PET membrane containing palladium (Pd) nanoparticles. They reported that these membranes could be used to purify H_2_ due to higher H2 permeability than those of O_2_, CH_4_ and CO_2._ [20].

In aqueous salt separation of polymeric films and membranes, Sum et al. investigated the amine-rich TFC membranes to separate heavy metals from aqueous solution. They reported that the rejections were in ascending order of Cd^2+^ <Cu^2+^ <Cr^3+^ [33]. Garcia et al found out that the diffusion rate (D) of KCl was 2 × 10^−7^ cm^2^ .s^−1^ across polyvinyl chloride (PVC)/graphene oxide based membranes while these membranes had 82%–97% rejection against KCl [26]. In another study, Meschke et al. studied the separation performance of commercial NF membranes and they observed that not only mono- and divalent ions but also uncharged solutes of some strategic elements such as Ge, Co, Mo were successfully separated from multicomponent aqueous solutions. They also reportedthat NaCl and MgSO_4_ rejections were found to be 85% and 97% through NE-90 membrane under NF conditions, respectively [34]. In a different study, Cheng et al. examined the diffusion dialysis performance of hybrid membranes of quaternary ammonium-polyvinyl alcohol (PVA) and tetraethoxysilane (TEOS). They reported that the prepared membrane showed much better separation factors against to HCl and FeCl_2_ than that of commercial DF-120 membrane [35]. As an alternative approach, layerby-layer (LbL) assembled ultrathin ion selective materials of cationic and anionic polyelectrolytes (PEs) were demonstrated [36–39]. In these studies, the multilayered membranes of different types of positively or negatively charged PEs were studied on their permeability for various metal chloride salts in aqueous solution. Tieke et al. showed that the membranes were permeable for sodium chloride, but much less permeable for divalent metal chlorides such as magnesium and zinc chloride, values for (NaCl/MgCl_2_) and (NaCl/ZnCl_2_) were found to be 43 and 20, respectively.

Concerning the selective separation of ions of polymeric membranes and films, there are some works reported under diffusion-dialysis, NF or RO conditions. Shintani et al. studied the effects of various monoamine addition during PA membrane and they found that all of these membranes showed efficient separation for sulphate (SO^2−^_4_) and magnesium (Mg^2+^) ions [29]. Similarly, Liu et al. prepared PA based membrane from piperazine and trimesoyl chloride (TMC) in the presence of additional diamine monomer for the purification of drinking water. These membranes displayed good selective separation of trace organic compounds and divalent cations in drinking water [40]. Kir et al. used poly(2-chloroaniline)/polyvinylidene fluoride (PVDF) composite membranes for the removal of chromium(III) and copper(II) ions from aqueous solution with Donnan dialysis. They reported that flux values of copper(II) ion were higher than chromium(III) because of hydration volume and charge of ions [41]. In a different study, Wen et al. reported that ion diffusion rates were in decreasing order of Li^+^ > Na^+^ > K^+^ > Cs^+^ > Mg^2+^ > Ca^2+^ > Ba^2+^ through PET film under 10 V applied voltage. It was also found that heavy metals such as Cu^2+^, Fe^2+^, Mn^2+^, and Cd^2+^ can hardly penetrate to the film [42]. In another work, ion-selective membranes prepared upon LbL assembly of azamacrocycles and PEs were reporte and high separation factors were achieved for α(Na+/Ca^2+^) and α(Na^+^/Mg^2+^) as 14 and 28, respectively [39].

Apart from the literature, it was aimed to examine and describe the selective mono- and divalent cation separation capability of economic/ commercially available polymeric films, namely polyethylene (PE) and polyamide6 (PA6), and commercial NF membranes from aqueous salt solutions in this study. For this purpose, the ion diffusion rates (D), separation factors (α) and ion rejections (R) of PE and PA6 films, and NF membranes were investigated comparatively as well as their morphology and wettability.

## 2. Experimental

### 2.1. Materials

High density polyethylene (HDPE) was purchased from Chevron Phillips Chemicals International (Woodlands, TX, USA) and polyamide6 (PA6) was supplied from EMS-Chemie Holding AG (Domat/Ems, Switzerland). They were used without further purification. NF90 (MWCO: 200 Da) and NF270 (MWCO: 400 Da) were supplied from Dow FilmTec (Dow Chemical Company, Midland, MI, USA) and used as commercial NF membranes. NaCl (purity ≥99.5%) and CaCl_2_ (purity ≥99.9%) were obtained from Sigma-Aldrich Chemie GmbH (Taufkirchen, Germany). Ethylenediaminetetraacetic acid (EDTA) and nitric acid (32.5%) were supplied from Sigma-Aldrich Chemie GmbH and their solutions were used as mobile phase to determine sodium ion in HPLC (high-performance liquid chromatography). Milli-Q ultrapure water (EMD Millipore Corp, Billerica, MA, USA) was used for the ion determination (resistance ≥18.2 MΩ cm^−1^) .

### 2.2. Preparation of polymeric films

**Figure 1:**
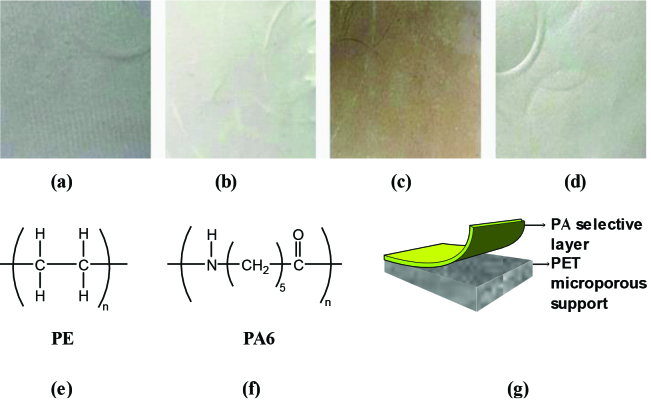
Pictures of (a) PE film, (b) PA6 film, (c) NF90, (d) NF270 and structures of (e) PE film, (f) PA6 film, and (g) commercial NF membranes (Deligöz et al.).

PE film was prepared by hot-pressing of solid PE pellets between two plates of PTFE (polytetrafluoroethylene) at 160° for 25 s under 20 bar. Same procedure was also used for the preparation of PA6 film at 198°. The thicknesses of all polymeric films were about 150 μ. The pictures of polymeric films [PE (Figure 1a), PA6 (Figure 1b)] commercial NF membranes [NF90 (Figure 1c), NF270 (Figure 1d)] and their (PE and PA6) chemical structures are given in Figures 1e and 1f, respectively. One can also see from Figure 1g that the NF membranes consist of two layers, polyamide (PA)-based selective top layer and polyether sulfone (PES) based porous support layer [43,44].

### 2.3. Ion diffusion experiments and separation performances

**Figure 2:**
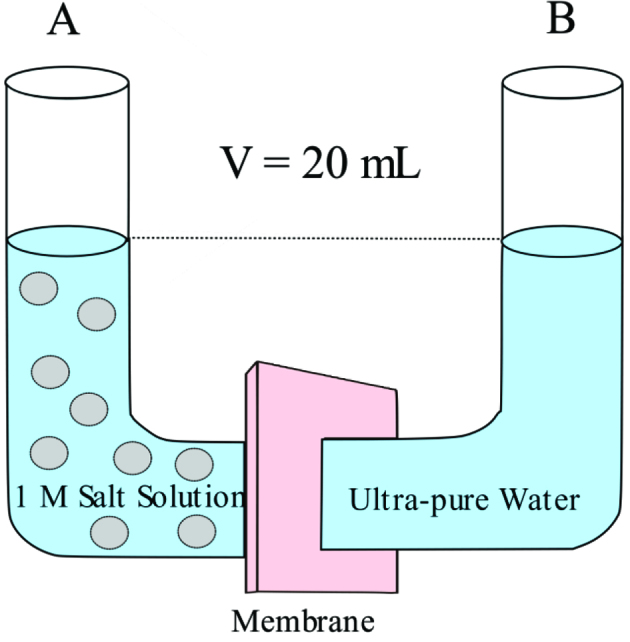
Schematic figure of U-type diffusion-dialysis system (Deligöz et al.).

Ion diffusion experiments were performed for the determination of separation performances of the polymeric films and NF membranes under diffusion-dialysis. For this purpose, U-shaped diffusion cell comprising two chambers containing 20 mL of salt solution (1M NaCl or CaCl_2_) and 20 mL of pure water was used as reported earlier [45].The set-up of home-made diffusion-dialysis system is shown in Figure 2.

This diffusion cell was used to investigate the diffusivity of mono-(Na^+^) and divalent cations (Ca^2+^) through polymeric films and commercial NF membranes for 1 h. Prior to the diffusivity measurements of different ions in the diffusion cell, the films were carefully washed several times with Milli-Q ultrapure water. Firstly, the diffusion rate of Na^+^ (0.1 M solution) was measured. Then the membrane was thoroughly cleaned and subjected to the second diffusion measurement with calcium chloride. The ion transport was followed by measuring Na^+^ or Ca^2+^ content in the permeate chamber. Agilent 1260 HPLC system with zorbax 300-SCX 4.6 mm ID×250 mm (5μm) column was used for sodium ion (Na+) determination. 0.1 mM EDTA/3 mM HNO solution were used as mobile phase. On the other hand, wet chemical method was used for the determination of diffused calcium ions (Ca^2+^) according to the literature [46]. Ion concentration measurements replicated twice, and it was found that the deviations were lower than 1%.

Subsequently D values for Na^+^ and Ca^2+^ were calculated according to the Equation 1 [47].

(1)CB(t)=A x D X CA0x ( t -t0)VB x L

where C_A0_ is the initial salt concentration in column, C_B(t)_ is the concentration of salt in column B, VB is the volume of liquid in column B, A, and L are the area and the thickness of film, respectively. D is the diffusion rate or diffusion coefficient ofions through the polymeric film or NF membrane (cm^2^.s^−1^) and (t-t_0_) is the difference between initial and final time in seconds. Please note that no correction for the osmosis was considered in order to observe the osmosis effect during diffusion-dialysis.

Furthermore, the separation factors (α) and rejections of mono- and divalent ions from polymeric films and commercial NF membranes were calculated from Equations 2 and 3 as shown below, respectively. α values of the films and commercial NF membranes were simply calculated by taking the ratio of diffusion rates of Ca^2+^ and Na^+^.

(2)α(Ca2+/Na+)=D(Ca2+)/D(Na+)

where D(Na^+^) and D(Ca^2+^) are the corresponding ion diffusion rates. On the other hand, the rejections of mono- and divalent ions were calculated from Equation 3.

(3)R=(1-CpCfx 100 

where, C_p_ and C_f_ are the concentrations of ions in the column B and column A, respectively [48].

### 2.4. Surface characterization

Scanning electron microscopy (SEM) and surface contact angle analyses were performed to examine the surface morphology and hydrophilicity of polymeric films and commercial NF membranes, respectively. SEM analyses were carried out with FEI Quanta FEG 450 Instrument while contact angle (θ) analyses were performed using KSV Attension THETA device. In SEM analyses, the polymeric films and commercial NF membranes were coated with nanothick gold layer prior to imaging and surface photographs of these samples were taken at 2000 and 10,000 magnification ratios under vacuum. In surface contact angle measurements, analyses were carried out by taking measurements from at least 3 points on the sample using 5 μL of water by pendant drop method.

## 3. Results and discussion

In this contribution, selective ion separation properties of polymeric films and commercial NF membranes under diffusion-dialysis conditions in terms of diffusivity, ion rejection and separation factor were studied comparatively.

### 3.1. Diffusivity and ion separation

In the study, Na^+^ and Ca^2+^ diffusivity through the polymeric films and commercial NF membranes were comparatively investigate dand the obtained results are given in Table. Further α values for Ca^2+^/Na^+^ are presented in the same table. As one can see from Table, Na^+^ diffusion rates for the films based on PE and PA6, and NF membranes were around 0.7–1.8×10^−8^cm^2^.s^−1^. Generally speaking, it can be said that the diffusion rates of Na+ through all samples were in the decreasing order of PE>NF90>NF270 and PA6. In addition to the diffusion rates of Na^+^, D(Ca^2+^) values were found to be ranging from 7.4×10^−8^ to 18.4×10^−8^cm^2^.s^−1^. If we compare the D(Ca^2+^) and D(Na^+^), it can be clearly stated that divalent salt was largely diffused through the polymeric films and membranes. 

**Table:** Diffusion rates (D), rejections (R) and α (Ca^2+^/Na^+^) for polymeric films and commercial NF membranes.

Film	D (Na^+^) cm^2^.s^−1^	D (Ca^2+^) cm^2^.s^−1^	Rejection (Na^+^)%	Rejection (Ca^2+^)%	α (Ca^2+^/Na^+^)	Reference
PE	1.8 × 10^−8^	7.4 × 10^−8^	99	96	4.1	This work
PA6	0.7 × 10^−8^	14.8 × 10^−8^	99.6	92	20	This work
NF90	1.5 × 10^−8^	14.8 × 10^−8^	99.2	92	10	This work
NF270	1.3 × 10^−8^	18.4 × 10^−8^	99.3	90	14.3	This work
GO	4.5 × 10^−8^	-	63	–	–	[59]
aza6/PVS	8.5 × 10^−11^	0.6 × 10^−11^	58	62	0.07	[39]
PAN/PET	3.7 × 10^−11^	1.5 × 10^−1^1	–	–	0.4	[60]

GO: Graphene oxide, aza6: Hexacyclen, PVS: Polyvinylsulfate, PAN: Polyacrylonitrile, PET: Polyethylene terephthalate.

**Figure 3:**
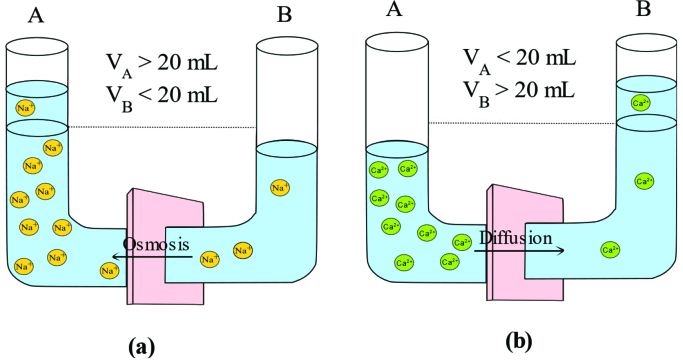
Schematic figures of (a) osmosis, and (b) diffusion across polymeric films and membranes (Deligöz et al.).

Indeed, it was supposed that the transport of monovalent ions can be fast compared to larger ones through the polymeric films or NF membranes [39,49]. However, we observed the opposite diffusivity results for monoand divalent ions regardless of the separator type. This lower D(Na^+^) values compared to D(Ca^2+^) can be explained by the higher chemical potential of CaCl_2_ than that of NaCl [50]. In other word, NaCl has lower osmotic pressure that may lead faster osmosis. Schematic representation of this phenomenon can be seen in Figure 3. Thus, osmosis can occur in the case of using monovalent salt solution while diffusion of divalent salt took place across the barrier dominantly. In the osmosis, the solvent (water) can move from a region of lower concentration to a region of higher solute concentration (Figure 3a) whereas diffusion can be described as the movement of solute molecules from an area of higher concentration to an area of lower concentration (Figure 3b). Thus, water can back diffuse to column A if NaCl was used and this can result in a decrease in the volume of column B. As explained above, no correction for the osmosis was considered in order to observe the osmosis effect during diffusion-dialysis. As it is purposed in Equation 1, the smaller volume in column B can yield a lowering in the diffusion rate of a corresponding salt since the volume of that column directly affect the diffusivity of salt. Therefore, an observable decrease in the diffusion rate of sodium ion through the polymeric films and membranes was detected.

**Figure 4:**
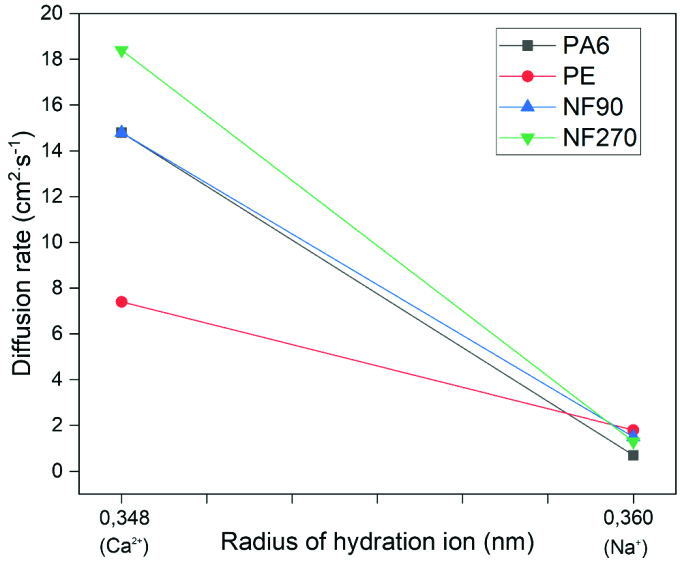
Diffusion rate changes of polymeric films and membranes depending on the sizes of hydrated ions (Deligöz et al.).

On the other hand, the other important factor determining the diffusivity is ion size in hydrated conditions as it is known from the literature [51,52]. In a study, Yang et al. investigated the effects of size of some hydrated ions and the ions channels selectivity on their transport properties. They reported that the transport properties of ions in aqueous solution should be strongly related to the average volume and hydration number of its hydrated ions. The sizes of hydrated sodium and calcium ions were reported as 0.360 nm and 0.348 nm while their ion sizes were found to be 0.098 nm and 0.106 nm in nonhydrated state, respectively. Figure 4 shows the diffusivity changes of polymeric films and NF membranes with the sizes of hydrated ions. As one can see from the figure that PE was slightly depended on the hydrated sizes of Na^+^ and Ca^2+^ ions whereas PA6 and NF270 were obviously affected. The less hydrated ion size dependency on diffusion coefficient of PE film may be corresponded to its crystalline structure and defect-free surface.

Among the diffusivity of ions presented in Table, there was an unexpected result showing the highest D(Na^+^) value for PE. Indeed, PE was supposed to exhibit better blockage to transport of aqueous salt solutions due to its relatively nonpolar character, high packing density, crystalline structure and defect-free surface. However, the opposite result was observed for D(Na^+^) through PE film. Most reasonable explanation can be the retarded osmosis through PE film due to its very dense structure (SEM figures of PE film confirm this). Moreover, the lowest D(Na^+^) for PA6 can be expressed in the similar way due to its relatively heterogeneous surface which might lead to form channels for osmosis. In addition, NF membranes displayed nearly the same diffusion rates for Na^+^. Concerning D(Ca^2+^) values, the expected trend was observed for all samples and the largest D(Ca^2+^) was found to be 18.4 × 10^−8^ cm^2^.s^−1^ for NF270 whereas the smallest diffusion coefficient was detected as 7.4 × 10^−8^cm^2^.s^−1^ for PE film. Probably this large diffusivity of NF270 can be corresponded to the relatively large porosity, and hence large molecular weight cutoff (MWCO) of this material. Comparing the obtained diffusion rates of ions under diffusion-dialysis with the literature as outlined in Table, it can be concluded the order of diffusion rates are comparable. On the other hand, the differences between diffusivities may be arisen from the type/structure of membranes and the technique to be used for ion content determination.

Besides, separation factors (α) for Ca^2+^/Na^+^ were found to be ranging from 4.1 to 20 in the increasing order of PE<NF90<NF270<PA6 (Table). The bigger separation factor was 20 for PA6 film whereas PE film showed the lowest separation factor (4.1). This reduced separation capability of PE can be corresponded to the similar ion diffusivity values due to the existence of osmosis during diffusion-dialysis supremely. Regarding the separation factor of NF membranes, it was pointed out that NF270 had a larger α value than that of NF90. This higher separation capability of NF270 can be attributed to more Ca^2+^ diffusion and less Na+ diffusion compared to NF90 due to its suitable pore size adjusting the permeabilities of mono- and divalent salt solutions. When compared to Tieke’s study [53], those results showed that PA6 film can be a promising separator for Ca^2+^ and Na^+^ due to its high separation capability as well as their controllable diffusion rates. Apart from the literature, it was determined that α values of (Ca^2+^/Na^+^) were bigger than that of (Na^+^/Ca^2+^) if th osmosis effect was considered during diffusion-dialysis.

### 3.2. Rejection of ions

The rejection of mono- and divalent ions from the polymeric films and NF membranes are calculated and given in Table. It was determined that Na^+^ rejection values of NF membranes and polymeric films exposed to 1M NaCl solution for 1 h ranged from 99% to 99.6%. It can be said that Na^+^ rejection values were very high and showed the similar trend to diffusion rate values. Accordingly, the lowest Na+ rejection was found for PE film while PA6 displayed the highest (99.6%) monovalent ion removal. This result for PA6 can be quite understandable due to the lowest NaCl diffusivity amongst all samples. Similarly, PE having large Na^+^ diffusion rate (1.8 × 10^−8^cm^2^.s^−1^) showed the worst Na+ rejection. This retarded osmosis for PE film may result in a relatively facilitated NaCl transport across PE film as explained above (Table). Concerning the commercial NF membranes, nearly the same Na^+^ rejections were determined around 99.2%–99.3%. Although it was expected that the rejection value of NF90 would be higher than that of NF270 considering their pore size, it was observed that NF membranes showed very similar Na^+^ rejection under diffusion-dialysis conditions. This may be explained by the osmosis occurrence during diffusion-dialysis.

In addition to Na^+^ rejections, Ca^2+^ rejection values were found to be ranging from 90% to 96%. The similar trend was observed for divalent ion rejection compared to monovalent ion. If we compare the ion rejections depending on the charge number of ions, it was observed that the rejection values were slightly bigger for sodium ion than that of calcium ion. This phenomenon can be explained by the fact that ion rejection reduced with hydrated ion diameter as explained above. The largest Ca^2+^ rejection was observed for PE film which had lowest diffusion rate for Ca^2+^ (7.4 × 10^−8^ cm^2^.s^−1^) whereas the lowest Ca2+ rejection value was seen for NF270 as 90%. This can be explained by the relatively larger porosity and MWCO of active layer that cannot efficiently block Ca^2+^ transfer. Another explanation is that rejection mechanism depends not only the sizes of ions but also surface charges of the NF membranes. It was found from the literature that different zeta potentials for NF90 (–59.27 ± 0.22 and –54.2 ± 2.7 mV) and NF270 (–82.08 ± 0.88 and -79.7 ± 1.6 mV) were reported [54–56]. Thus, the attraction forces between negative charges of the NF membranes and the positive charges of cation could facilitate its transport across the active layer by limiting the electrostatic repulsion [56]. In summary, one can conclude that the ion rejections of polymeric films and NF membranes are comparable with the literature [31,57] for diffusion-dialysis system regardless ion charge of the solution.

### 3.3. SEM (scanning electron microscopy) analysis

**Figure 5:**
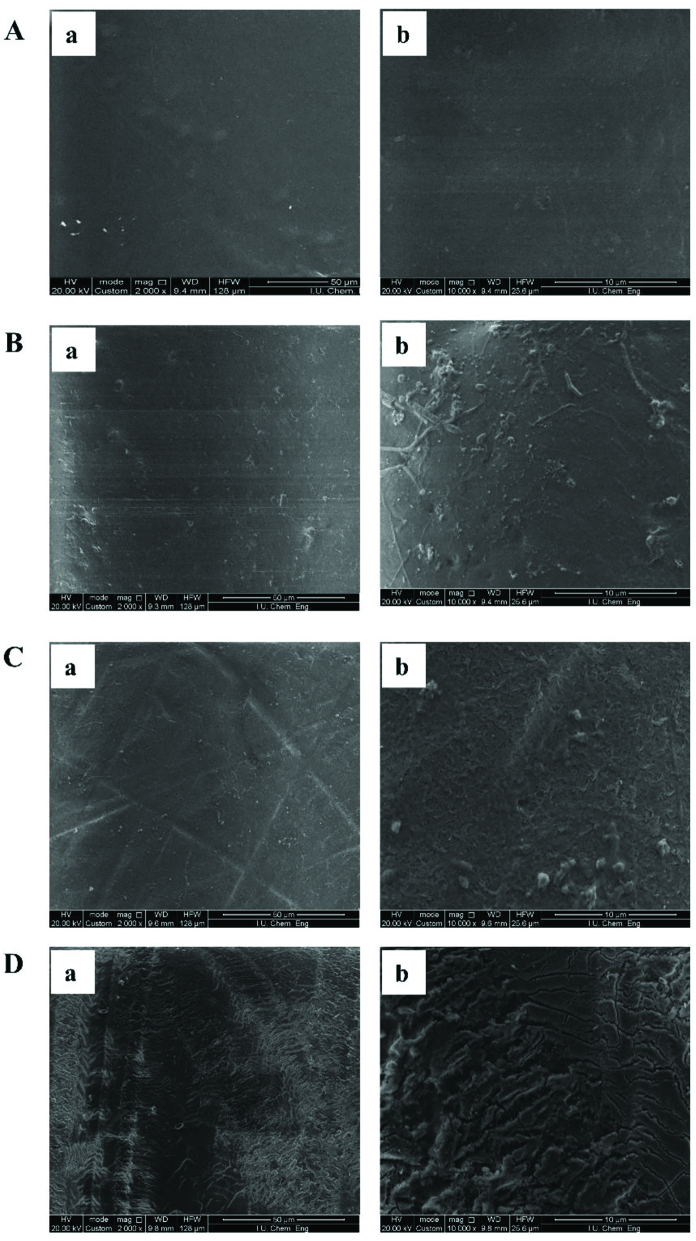
SEM images of polymeric films and commercial NF membranes at (a) 2000, and (b) 10,000 magnifications for (A) PE film, (B) PA6 film, (C) NF90, (D) NF270 (Deligöz et al.).

To determine the morphology of polymeric films and commercial NF membranes, SEM images were taken from the surfaces at 2000 and 10,000 magnification ratios. The obtained pictures are shown in Figure 5. One can see from Figures 5Aa and 5Ab that PE film had completely dense, nonporous and defect-free surface while PA6 film exhibited nonporous surface with some heterogeneous formations (Figures 5Ba and 5Bb). At 10,000 magnification, these heterogenous appearances became more obvious and it was evaluated that these formations may be attributed to the unmelted polymers during hot-pressing. In other word, PE film had more uniform and homogenous surface than that of PA6 film. Probably the reason is applying enough temperature to melt PE completely while the temperature range may be not sufficient to melt PA6 completely. In SEM images of the commercial NF membranes, NF90 displayed relatively dense, defect-free and homogenous surface (Figure 5Ca and 5Cb) whereas some formations like fibrils on the surface of NF270 can be seen at bigger magnification ratios. These fibrillose structure (Figure 5Da and 5Db) can be corresponded to the supporting material.

### 3.4. Contact angle measurements

**Figure 6:**
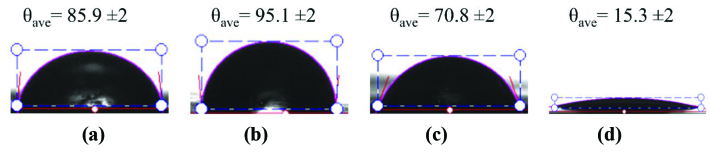
Contact angle images of (a) PE film, (b) PA6 film, (c) NF90, and (d) NF270 (Deligöz et al.).

In order to investigate the hydrophilicity of polymeric films and NF membranes, contact angle (θ) analyses were performed and the obtained pictures are given in Figure 6. As it is known from the literature, not only the chemical structure but also surface morphology/topography of a material strongly affect *θ* values [58]. That is why, the following results were discussed considering above explanation. The average contact angles ( *θ_ave_* ) of PE (Figure 6a) and PA6 (see in Figure 6b) were found to be 85.9 ± 2 and 95.1 ± 2, respectively. Indeed, the lower θave would be expected for PA6 due to its relatively more polar structure comparing PE, but the opposite result was observed. The high θave for PA6 may be explained by its surface heterogeneity shown in SEM figures owing to possibly unmelted PA6 during the film processing. Besides, the commercial membranes displayed *θ_ave_* as 70.8 ± 2 and 15.3 ± 2 for NF90 (Figure 6c) and NF270 (Figure 6d), respectively. The lowest *θ_ave_* of NF270 can be explained by the surface morphology. As it is known from the literature that NF commercial membranes (especially NF270) has some nanoscaled porosities on the active layer to enhance flux properties while they were supported by a porous material [30]. Although, we did not observe any porosity on the surface of NF membranes in the range of magnification ratios studied, the changes in nanoscale may lead to control the surface hydrophilicity. In conclusion, θave of polymeric films exhibited less hydrophilic character whereas NF membranes had larger hydrophilicity.

## 4. Conclusions

In this contribution, selective separation of mono- and divalent ions through polymeric films and NF membranes were studied and discussed comparatively. For this purpose, PE and PA6 films prepared by hot-pressing and 2 different NF membranes, namely NF90 and NF270, were used. The prepared films and commercial NF membranes were studied on their diffusivities (D), rejections (R) and separation factors(α) for NaCl and CaCl_2_ in aqueous solution as well as their morphology and hydrophilicity.

It was found out that the diffusion rates of the ions were in the order of 10^−8^cm^2^.s^−1^. The membranes were permeable for calcium chloride, but less permeable for sodium chloride, the theoretical separation factor (CaCl_2_/NaCl) was 20 for PA6 film. D, R, and α of the polymeric films and commercial membranes can be strongly affected by the presence of diffusion and osmosis processes and the sizes of hydrated sodium and calcium ions. It was also found that the diffusion was more favourable for CaCl_2_ transport, osmosis occurred for NaCl due to its low chemical potential. On the other hand, the hydrated sodium and calcium ions (around 0.3–0.4 nm) can be selectively passed through the polymeric films and NF membranes. Expectedly, the ion diffusivity became smaller with hydrated ion diameter. Larger hydrated size of Na^+^ may also explain lower D and higher R. In conclusion, it was evaluated that PA6 may be a good alternative for selective separation of mono- and divalent cations due to relatively its high separation factor (α = 20) among the studied samples.
